# Influence of the Lipid Backbone on Electrochemical Phase Behavior

**DOI:** 10.1021/acs.langmuir.2c02370

**Published:** 2022-11-10

**Authors:** Philip N. Jemmett, David C. Milan, Richard J. Nichols, Thomas Howitt, Alexandra L. Martin, Thomas Arnold, Jonathan L. Rawle, Christopher L. Nicklin, Timothy R. Dafforn, Liam R. Cox, Sarah L. Horswell

**Affiliations:** † School of Chemistry, University of Birmingham, Edgbaston, BirminghamB15 2TT, UK; ‡ Department of Chemistry, University of Liverpool, Crown Street, LiverpoolL69 7ZD, UK; § Diamond Light Source, Harwell Science and Innovation Campus, Chilton, Didcot, OxfordshireOX11 0DE, UK; ∥ European Spallation Source ERICPO Box 176, LundSE-221 00, Sweden; ⊥ ISIS Pulsed Neutron and Muon Source, Science and Technology Facilities Council, Rutherford Appleton Laboratory, Harwell, OxfordshireOX11 0QX, UK; # Department of Chemistry, University of Bath, Claverton Down, BathBA2 7AY, UK; ∇ School of Biosciences, University of Birmingham, Edgbaston, BirminghamB15 2TT, UK

## Abstract

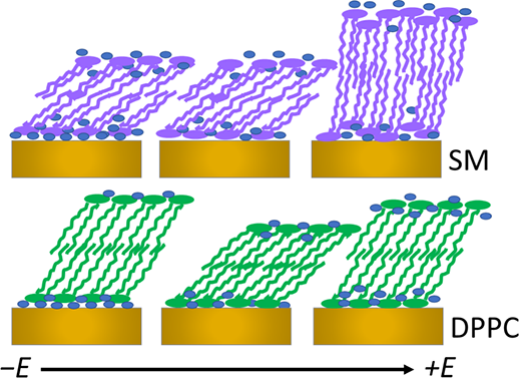

Sphingolipids are an important class of lipids found in mammalian cell membranes with important structural and signaling roles. They differ from another major group of lipids, the glycerophospholipids, in the connection of their hydrocarbon chains to their headgroups. In this study, a combination of electrochemical and structural methods has been used to elucidate the effect of this difference on sphingolipid behavior in an applied electric field. *N*-Palmitoyl sphingomyelin forms bilayers of similar coverage and thickness to its close analogue di-palmitoyl phosphatidylcholine. Grazing incidence diffraction data show slightly closer packing and a smaller chain tilt angle from the surface normal. Electrochemical IR results at low charge density show that the difference in tilt angle is retained on deposition to form bilayers. The bilayers respond differently to increasing electric field strength: chain tilt angles increase for both molecules, but sphingomyelin chains remain tilted as field strength is further increased. This behavior is correlated with disruption of the hydrogen-bonding network of small groups of sphingomyelin molecules, which may have significance for the behavior of molecules in lipid rafts in the presence of strong fields induced by ion gradients or asymmetric distribution of charged lipids.

## 1 Introduction

Biological cell membranes perform a crucial role in a cell, forming a selective barrier that allows the cell to regulate the transport of ions and molecules between the extracellular fluid and cytosol or between different compartments of a cell.^1^ The basis of the membrane is a lipid bilayer containing a variety of receptors, signaling molecules, and functional proteins. There is a wide variety of lipids in natural cell membranes, and the membrane composition varies not only between species but also between cell types in an organism and between different membrane types within a cell.^1,2^ The lipid components of the bilayer have multiple structural and functional roles, which are now beginning to be appreciated.^3,4^ For example, the composition modulates local membrane tension, which can be important for the action of proteins,^5^ and some lipids have specific functional group interactions with proteins,^6−8^ while some are involved in cell-signaling processes.^3^ The lipid bilayer is a complex mixture of saturated and unsaturated lipids, lipids of different headgroups, and sterols. Its composition is heterogeneous: lipids are distributed asymmetrically across the two halves of the bilayer, and lateral phase separation can also occur, resulting in regions of differing physical properties.^1−3,9^ Regions or domains, known as ″lipid rafts″, enable colocalization of membrane proteins and are thought to be involved in trafficking and signal transduction.^3^ While there has been some debate over the nature of lipid rafts,^10,11^ it is generally accepted that they form at least transiently.^11,12^ The rafts are enriched in sphingolipids, which include glycosphingolipids, ceramides, and sphingomyelins.^10,13,14^

Sphingomyelins make up ∼15% of the outer half of mammalian cell membranes^15^ and are present in high quantities in brain tissue, red blood cells, and the lens of the human eye.^16^ They play multiple roles: they have a key structural role in the membrane,^16,17^ they are involved in endocytosis and receptor-mediated ligand uptake,^18^ they have been shown to have specific interactions with membrane proteins,^8^ and they act as a source of ceramide, which is produced under stress conditions and acts as a signal to initiate apoptosis.^13,17,19^ Their strong interaction with cholesterol has been suggested to play a role in inhibiting cholesterol absorption in the digestive tract and the properties they confer on the cell membrane to protect hepatocytes from the detergent action of bile salts.^20^

**Figure 1:**
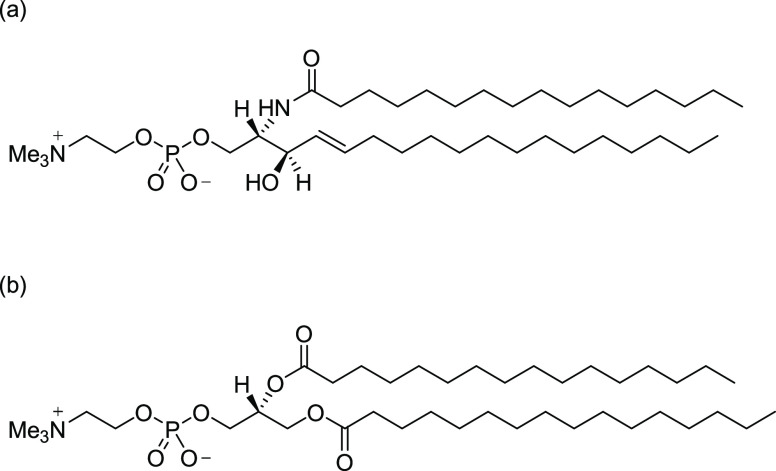
Molecular structures of (a) *N*-palmitoyl sphingomyelin (SM) and (b) di-palmitoyl phosphatidylcholine (DPPC).

Sphingomyelins differ from the other major group of membrane lipids, the glycerophospholipids, in the linkage of the fatty acid tails to the headgroup. Figure 1 compares the structures of *N*-palmitoyl sphingomyelin (SM) and di-palmitoyl phosphatidylcholine (DPPC). Both lipids have a palmitoyl chain and the same headgroup, phosphorylcholine. In DPPC, the headgroup is connected to a glycerol moiety to which the fatty acid tails are connected with ester groups, whereas in SM, the headgroup is connected to a sphingosine backbone containing a *trans* double bond and a hydroxyl group, and the second tail is connected via an amide linkage. These structural differences enable different intermolecular interactions between the molecules in a membrane. Whereas DPPC has only hydrogen bond acceptors in the phosphate and ester groups—and ester groups tend to be weaker hydrogen bond acceptors than amide groups—SM has several hydrogen bond acceptors and donors: the amide group, hydroxyl group, and phosphate group. These features enable strong interactions between SM molecules,^21,22^ which can result in strong mechanical properties and low permeability. Structural studies have shown that the average area per molecule and the chain tilt are smaller within SM multilayers (in the gel phase)^23^ and monolayers^24^ than for similar phosphatidylcholine layers, and recent molecular dynamics and spectroscopic studies have demonstrated the ability of SM molecules to form a variety of hydrogen bonds with each other as well as with water.^25−27^

Natural cell membranes are exposed to strong electric fields, which arise through charge asymmetry across the membrane and ion gradients across the membrane.^28^ It is important to understand the effect of these fields on membranes because they can induce a change in structure and, in some instances, breakdown of the membrane.^29^ These fields can be simulated in electrochemical experiments,^29,30^ and their effects on lipid monolayers have been determined in this way.^31−37^ By supporting a lipid monolayer or bilayer on an electrode, electrochemistry can be combined with structural and spectroscopic methods to gain insight into the effect of these fields on the structure of membranes.^29,30,38−56^ This approach has been used successfully to study the electrochemical phase behavior of phospholipids,^29,30,38−46^ their field-dependent interaction with other molecules,^47−51^ and the effect of structural differences of lipid molecules on their packing and response to an applied field.^52−56^ Given the pivotal role of sphingomyelins in lipid rafts and the fact that they are in proximity to ion channel proteins, we sought in this study to determine whether and how sphingomyelin differs from glycerophospholipids in its response to an applied field. We show that *N*-palmitoyl sphingomyelin (SM) bilayers have similar coverage on Au(111) to DPPC bilayers and that SM undergoes a phase transition upon increasing the strength of the applied field, which results in a change in chain orientation and hydration of the bilayer. IR spectra indicate that the phase transition in SM bilayers involves the breaking of direct hydrogen bond interactions between SM molecules, which are replaced with interactions with water molecules. Like DPPC, this effect appears to originate in the interaction of water with the field as the field strength increases but, unlike DPPC, the result is a different orientation of the hydrocarbon chains either side of the electrochemical phase transition. The implication is that the effects of polarization on sphingolipid-rich domains of a cell membrane may differ from those on other regions of the cell membrane, thereby influencing the local environment for functional molecules situated within those domains and the activity of the sphingolipids themselves.

## 2 Experimental Section

### 2.1 Materials

Ultrapure water (purified with a Millipore tandem Elix-MilliQ Gradient A10 system (resistivity ≥18 MΩ cm, TOC ≤ 5 ppb)) was used throughout. Glassware was cleaned with piranha solution (Caution! Can cause explosion!) or by heating in a ∼1:1 mixture of concentrated sulfuric and nitric acids for at least 1 h. In each case, the acid treatment was followed by thorough rinsing with copious quantities of ultrapure water and soaking in ultrapure water overnight. Viton O-rings, PTFE, and Kel-F parts were cleaned by soaking in a ∼1:1 mixture of hydrogen peroxide (30%) and ammonia (25%) solutions for several hours and then thorough rinsing with and soaking in ultrapure water overnight. The components of the spectro-electrochemical cell were dried in a designated clean oven prior to cell assembly.

Lipid solutions were prepared in a mixed solvent comprising 1 part methanol to 2 parts chloroform (by volume). Both solvents were HPLC grade (Sigma Aldrich). The lipids used in this work, *N*-palmitoyl sphingomyelin (SM) and di-palmitoyl phosphatidylcholine (DPPC), were purchased from Avanti Polar Lipids and used as received. Electrolyte solutions were prepared from sodium fluoride (Premion-grade, Alfa) and were made up as 0.1 M aqueous solutions.

### 2.2 Langmuir Trough Measurements

A Teflon Langmuir trough (Nima, UK), equipped with a dipper and Delrin barrier, was used for Langmuir isotherm measurements and for depositing lipid bilayers on gold substrates. The trough was prepared by cleaning with chloroform, and water was used as the subphase. The surface of the water was checked for cleanliness by monitoring the surface pressure over the entire available area (100–600 cm^2^). Seventy microliters of a 1 mg mL^–1^ solution of SM was deposited on the water surface using a microliter syringe, and the organic solvent was allowed to evaporate. Isotherms were recorded at a barrier speed of 25 cm^2^ min^–1^. A typical isotherm is given in the Supporting Information (Figure S1). The limiting area per molecule, obtained by extrapolating the *L*_c_ part of the isotherm to the abscissa, is ∼43 Å^2^. This value is within the expected experimental error of that obtained by Li et al. for C_16_ SM^57^ and is smaller than that measured for DPPC in our laboratory under the same conditions (∼50 Å^2^). This result shows that SM is more closely packed than DPPC in the monolayers. Y-type lipid bilayers were formed on clean gold electrodes or gold-on-glass slides using Langmuir–Blodgett deposition followed by Langmuir–Schaefer (horizontal touch) deposition (LB-LS deposition). The cleaned gold substrates were placed in the water subphase, and a lipid monolayer deposited on the surface of the water. One compression cycle was performed, and the monolayer was then compressed to a surface pressure of 40 mN m^–1^. The substrate was then withdrawn vertically through the interface at controlled surface pressure at a rate of 2 mm min^–1^ and then dried in argon for 30 min before the Langmuir–Schaefer deposition was performed. At this surface pressure, the SM lipid monolayer is in the L_c_ phase, and the area per molecule is ∼36–37 Å^2^.

### 2.3 Grazing Incidence X-ray Diffraction and X-ray Reflectivity Measurements

X-ray reflectivity (XRR) and grazing incidence X-ray diffraction (GIXD) measurements were carried out at the I07 beamline at Diamond Light Source (Oxfordshire, UK).^58^ X-rays (12.5 keV, λ = 0.9919 Å) were directed onto the water surface with a double crystal deflector system.^59^ A large-area (700 cm^2^) Langmuir trough (Nima) with temperature control was employed and encased in a box with a He atmosphere to reduce background scattering and beam damage. XRR data were collected over a *q_z_* range of 0.018 to ∼0.6 Å^–1^ and were reduced and corrected for footprint over-illumination with an in-house Python script. GIXD was measured with an angle of incidence corresponding to *q_z_* = 0.018 Å^–1^ and a pinhole geometry,^60^ allowing the acquisition of diffraction images, which were subsequently spliced to produce an image over a *q_z_* range of 0.0–0.8 Å^–1^, using an in-house-written MATLAB script. GIXD data were integrated and fitted using MATLAB scripts and OriginPro. Reflectivity data were fitted using a Bayesian MCMC algorithm within RasCAL_2019.^61^

### 2.4 Electrochemical Measurements

Electrochemical measurements were carried out in an all-glass three-electrode cell, which was connected to a reference electrode compartment through a salt bridge. A saturated calomel electrode (SCE, Hach Lange GmbH) was used as the reference electrode, and a gold coil (99.999%, Alfa Aesar) was used as the counter electrode. The counter electrode was cleaned by flame annealing and quenching with ultrapure water. The working electrode was a Au(111) single crystal, oriented to better than 0.5° (MaTecK GmbH, Germany). It was cleaned by flame annealing as described in the literature^62^ and transferred to the electrochemical cell with a drop of ultrapure water. NaF (0.1 M) was used as the electrolyte and was deoxygenated in the cell by bubbling with argon gas. An argon atmosphere was maintained above the solution throughout the experiment, and the electrochemical response of the clean system was checked before bilayer deposition.

Differential capacitance experiments were performed with a Heka PGSTAT590 potentiostat and a DSP7265 lock-in amplifier (Ametek). A potential sweep rate of 5 mV s^–1^ was used with an AC frequency of 20 Hz and an amplitude of 5 mV. The data were collected with in-house-written software via a data acquisition board (National Instruments). The software used to acquire the data was kindly provided by Dr. Alexei Pinheiro (Universidade Tecnologica Federal do Parana, Londrina, Brazil). The same software was used to control the potentiostat and record the data for chronocoulometry measurements. These measurements consisted of applying a series of potential steps, as described in previous publications.^29,52^ Briefly, the potential was held at a base potential of −0.01 V (vs Ag|AgCl|3 M KCl) before being stepped to the potential of interest. It was maintained at this potential for 3 min to allow equilibrium to be established and then stepped for 0.15 s to a potential that is sufficiently negative to desorb the lipids (−0.91 V) before being returned to the base potential for 1 min. A current transient was recorded during the desorption step. This process was repeated for a series of potentials, stepping in increments of 0.05 V in the negative direction. Each current transient was integrated to give the total charge passed during the potential step. These relative charge densities were then converted to absolute charge densities using the potential of zero charge of the bare electrode.

### 2.5 Atomic Force Microscopy

SM bilayers were deposited on 11 mm × 11 mm gold-on-glass slides (Arrandee, Westfalen, Germany) for AFM measurements. These glass slides consist of glass coated with an ultrathin chromium adhesion layer and a ∼250 nm layer of gold. The slides were briefly flame-annealed, cooled, and transferred with a drop of water to the Langmuir trough for deposition. After flame annealing, the surface consists of reconstructed (111) microcrystalline regions.^63^ AFM measurements were performed with a Nanoscope IIIA (Digital Instruments). Measurements were carried out in tapping mode in air using Au NT-MDT cantilevers (CSG30, Golden Silicon Probes). The nominal resonant frequency was 48 kHz, which was then calibrated to find the AC tuning resonant frequency of 39 kHz. The nominal force constant of the tips was 0.6 N m^–1^, and the cantilever-tip assemblies were calibrated to give a value of 0.308 N m^–1^. Images were flattened and plane-fitted as required using Gwyddion software.^64^ Gwyddion was also used to measure profiles across defects on the images to determine the bilayer thickness.

### 2.6 Infrared Measurements

Polarization-modulated infrared reflection absorption spectroscopy (PM-IRRAS) measurements were carried out with a Bruker Vertex 80v spectrometer equipped with an external PMA50 module. The PMA module includes a photoelastic modulator (PEM-100, Hinds Instruments, US) with a ZnSe 50 kHz optical head and a synchronous sampling demodulator (GWC Technologies, US). The data were acquired at a resolution of 2 cm^–1^ with a liquid nitrogen-cooled MCT detector. Spectra were measured at 19 °C (±1 °C), at which temperature SM is in the gel phase.^23^ Transmission spectra were also measured to enable the calculation of isotropic optical constants of SM in H_2_O and in D_2_O, which are needed to calculate the orientations of transition dipole moments in the PM-IRRA spectra (vide infra).^65^ These spectra were acquired using a demountable liquid cell (PIKE Technologies, Madison, US) with a 25 μm spacer and using BaF_2_ windows. The spectra were acquired using 0.1 M NaF (in H_2_O or D_2_O) solutions for both the analyte solution and background solution. NaF was used to suppress dissolution of the BaF_2_ windows. Optical constants were calculated using software kindly provided by Dr. Vlad Zamlynny (Acadia University, Canada).^66^

A custom-built cell was used to perform the spectroelectrochemical measurements. The cell window was a CaF_2_ 1″ or BaF_2_ 1″ equilateral prism (Crystran, UK). The working electrode, on which the lipid bilayer was deposited, was a Au(111) single crystal (99.999% purity, orientation < 0.5°, MaTecK, Germany). The counter electrode was a gold coil (99.999%, Alfa Aesar) and was arranged concentric to the working electrode. The Ag|AgCl|3 M KCl reference electrode (BASi, US) was connected to the cell via a tube placed as close to the working electrode as possible. All potentials in this work are reported with respect to the Ag|AgCl electrode. The electrolyte used in the cell was 0.1 M NaF (Premion-grade, Alfa Aesar), again to suppress dissolution of the BaF_2_ window. 0.1 M NaF was made up in ultrapure water for investigating the phosphate stretching region or in deuterium oxide for investigating the C–H and C=O stretching regions.

The PEM was set for half-wave retardation at 2900 cm^–1^ for the C–H stretching region, at 1600 cm^–1^ for the C=O stretching region, and at 1100 cm^–1^ for the phosphate stretching region. The signal obtained is dependent on the angle of incidence of the infrared light and the thickness of the electrolyte layer between the sample and the window. The optimum values of angles of incidence and gap thickness were chosen using the values calculated by Zamlynny.^67^ For a Au surface in these electrolytes, these values are 51° and 2 μm (BaF_2_) or 57° and 3 μm (CaF_2_) at 2900 cm^–1^, 67° and 3.0 μm (CaF_2_) at 1600 cm^–1^, and 57° and 2 μm (BaF_2_) at 1100 cm^–1^. Thicknesses were calculated by comparing the reflectivity spectra with a simulated reflectivity spectrum for the cell configuration.^65^ This procedure was carried out with “Fresnel 1” software kindly provided by Dr. Vlad Zamlynny.^66^

## 3 Results and Discussion

### 3.1 Grazing Incidence X-ray Diffraction and X-ray Reflectivity Measurements

**Figure 2:**
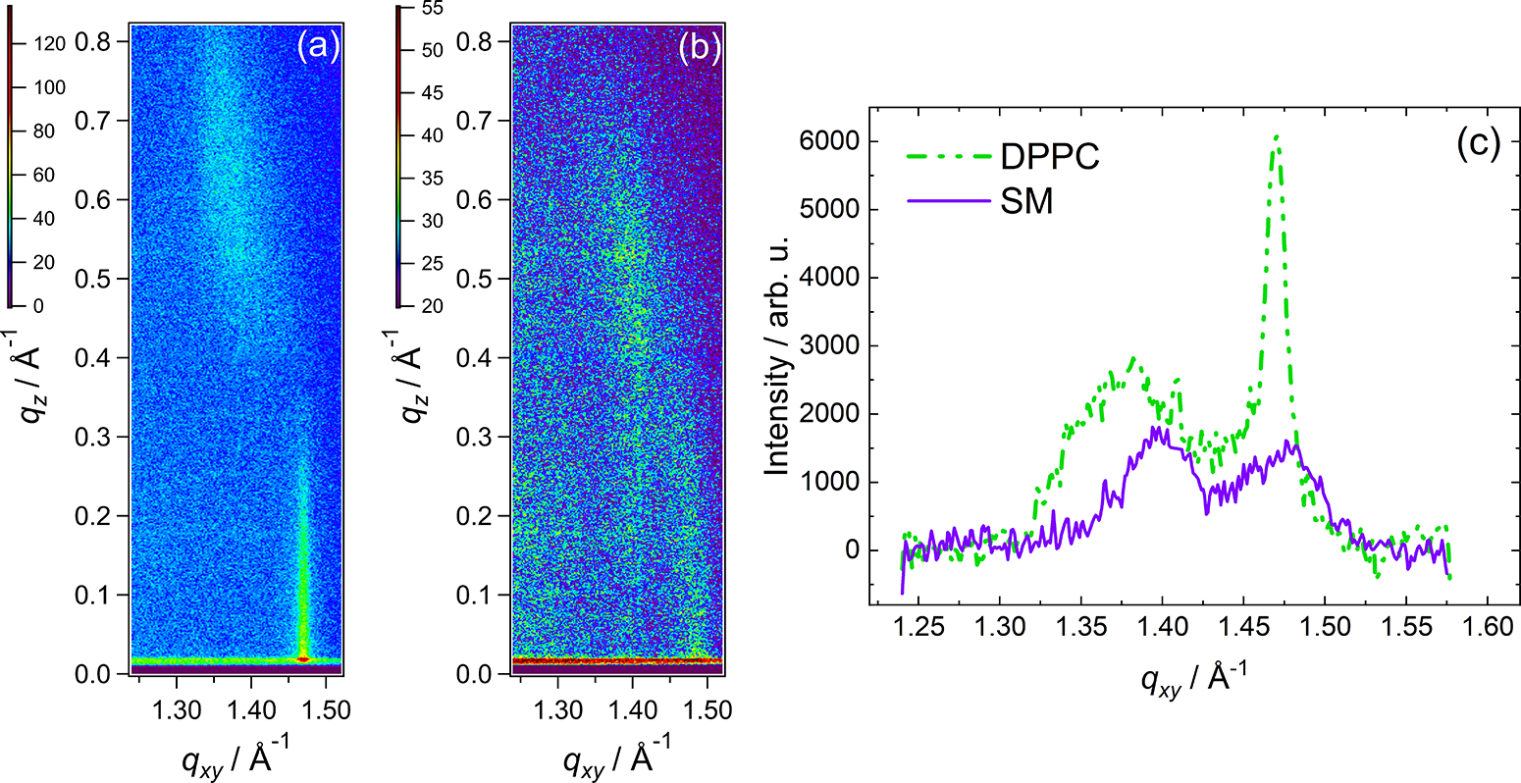
(a) GIXD image of DPPC. (b) GIXD image of SM. (c) Resulting plots of the integrated intensity vs *q_xy_* for DPPC and SM.

GIXD and XRR were carried out on monolayers at the air|water interface to aid the interpretation of PM-IRRAS results. Figure 2 presents GIXD images of the DPPC and SM monolayers at 40 mN m^–1^, the pressure at which the supported bilayers were deposited. The DPPC images are consistent with literature reports.^68^ The diffraction peaks arise from the scattering of X-rays from the hydrocarbon chains of the molecules, which are packed in an ordered arrangement at this surface pressure. The positions of the peaks can be analyzed to deduce information on the packing arrangement, unit cell area, and chain tilt angle.^69^ The presence of two peaks for DPPC, one of which is located at *q_z_* ≈ 0, indicates that DPPC molecules have chains tilted in or close to the nearest neighbor direction. The images acquired for SM differ from those of DPPC; SM also gives two peaks but at closer values of *q_z_*, showing that the chains of SM molecules are packed in a different arrangement, with chains tilting in a different in-plane direction. The intensity of the SM peaks is lower than that of the DPPC peaks, and the {1̅ 1} reflection is broader, indicating that the degree of ordering of SM is lower despite the slightly closer packing (vide infra). It is likely that the multiple possibilities for hydrogen bonding between SM molecules result in a less coherent arrangement of molecules and smaller crystalline domains than in DPPC, where the intermolecular interactions are dominated by chain packing.

The intensity integrated over *q_z_* 0.0 to 0.7 Å^–1^ is plotted against *q_xy_* in Figure 2c. The peaks in Figure 2c were fitted to a Voigt function and the positions used to calculate the area occupied by each chain and thence the area per molecule. The resulting parameters for DPPC and SM are given in Table 1. The *q_z_* positions of the Bragg rods were also obtained from the data and used, along with the *q_xy_* positions, to calculate the tilt angle, θ, of the hydrocarbon chains from the surface normal, using eq 1:^70^
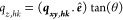
1where ***ê*** is the unit vector pointing along the chain tilt direction. The tilt angles are also included in Table 1. Note that the peak at lower *q_xy_* appears to be formed from two broad peaks that are difficult to resolve. Fits treating this peak as one peak or as two peaks yield similar area per molecule and tilt angle from the surface normal, so for simplicity, the results using one peak in the fit are reported here. The results for DPPC are in good agreement with those reported by Watkins et al. for DPPC monolayers at the same surface pressure.^68^ The tilt angle obtained for the SM chains is smaller than for DPPC, around 21° for SM compared with 27° for DPPC. Below, we shall see that the tilt angle of an SM bilayer on Au(111) is also lower than that of DPPC at low charge densities. The smaller area per molecule obtained for SM results from the smaller tilt angle of the hydrocarbon chains but the difference in molecular areas between DPPC and SM is smaller than that observed in the isotherm measurements. The longer-chain sphingomyelin (C_18_ chain) has been reported to give diffraction with one broad Bragg peak, which indicates that it forms monolayers of untilted chains with little lateral ordering.^71^ The C_16_ SM studied in the present work may have slightly tilted chains to increase dispersion interactions between chains as the chains occupy smaller areas than the headgroups—the longer chain C_18_ molecule would need to tilt less to compensate for the difference in footprint. Egg-SM, on the other hand, has been reported to produce two peaks,^72^ very similar to the result in Figure 2, which suggests that the ordered domains in egg-SM are dominated by *N*-palmitoyl SM (which constitutes approximately 80% of the mixture^73^).

**Table 1:** Unit Cell Parameters Derived from Fitting the GIXD Data^a^

	*a*, *b* (Å)	γ (°)	*A*_M_ (Å^2^)	θ (°)	ψ (°)
DPPC	5.054 (0.030)	116.1 (0.0003)	45.89 (0.40)	27.1 (0.7)	<7
SM	5.013 (0.046)	116.2 (0.01)	45.08 (0.16)	20.6 (2.0)	12 (4)

a Numbers in brackets represent estimated errors using standard deviations of four separate measurements.

XRR data for DPPC and SM are presented in the Supporting Information (Figure S2). The XRR curves are similar and therefore indicate little difference in electron density profiles between the two monolayers at this surface pressure within the error of the measurement or the fit. The data were fitted to a model comprising a tail slab and a headgroup slab, with the roughness constrained to be the same between each slab interface. The fits for DPPC are, within error, consistent with those in a neutron study of DPPC at 35 mN m^–1^,^74^ although the interfacial roughness was higher in our measurement. However, XRR data acquired at 23 °C for DPPC at pressures above 40 mN m^–1^ have been fitted with similar roughness values to ours but slightly greater thickness.^75^ For SM, studies of egg-SM have been reported at a surface pressure of 25 mN m^–1^, where the tail group slab is thinner, partly a result of greater chain tilt angle and partly because of the presence of unsaturated lipids. Our monolayers are also slightly denser than those of egg-SM at the lower pressure, as expected for the higher surface pressure and a sample with all saturated chains. C_18_ SM XRR data have been reported and fitted to a three-slab model, which was designed to account for the staggering of lipid molecules in the *z*-direction that results from multiple hydrogen bonding interactions.^71^ As our data could be fitted to two slabs, albeit with higher roughness, these fits were chosen in preference as they represented the simplest model that could fit our data. The closeness in tail group slab density for SM and DPPC in our data is expected from the similar molecular areas determined in diffraction measurements, and the difference in chain tilt angle is not sufficient to result in a significant difference in slab thickness. The headgroup SLD and thicknesses are also close in value but, because the scattering lengths of the unsolvated headgroups differ and the molecular area of SM is slightly smaller, a slightly smaller headgroup volume (and an extra 0.8 water molecule per lipid) was derived for SM than for DPPC, although this difference is of the order of the error in the fit. The overall monolayer thicknesses are ∼25 Å, and the average number of water molecules per lipid headgroup is 2–3.

### 3.2 Electrochemical Measurements

**Figure 3:**
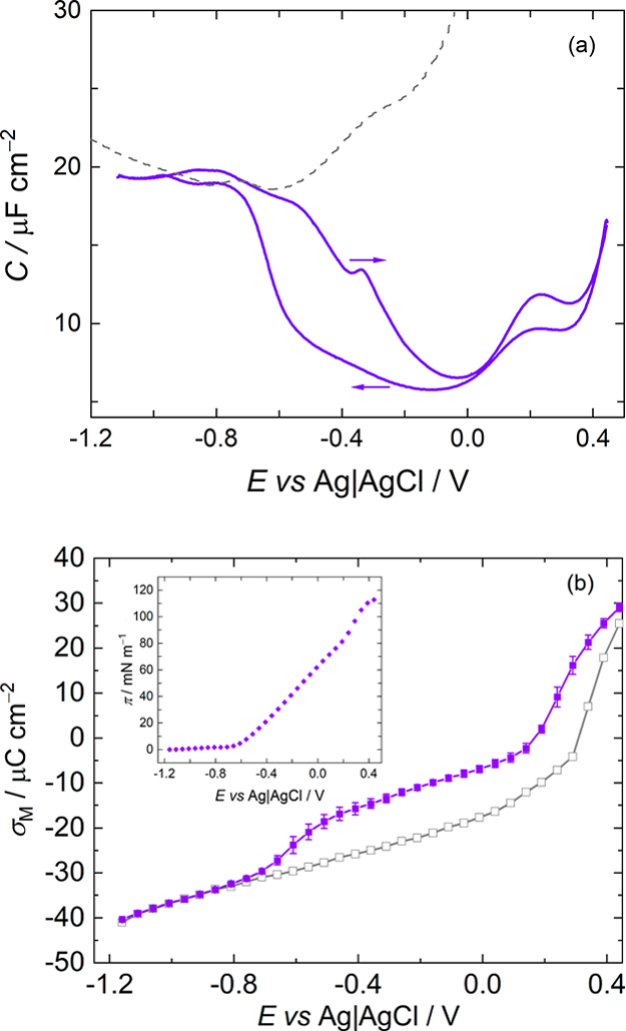
(a) Differential capacity of a Au(111) electrode (dashed line) and a Au(111) electrode coated with SM (solid line) in 0.1 M NaF. Sweep rate, 5 mV s^–1^. (b) Chronocoulometry data of a Au(111) electrode (open shapes) and a Au(111) electrode coated with SM (filled shapes) in 0.1 M NaF. Inset: surface pressure derived from the chronocoulometry data.

Figure 3 shows the differential capacitance of the interface between a Au(111) surface coated in a bilayer of SM and a 0.1 M NaF electrolyte. At negative potentials, the bilayer is desorbed from the electrode surface and the capacitance curve merges with that of the base electrolyte. At more positive potentials, the specific capacitance is lower in the presence of the bilayer, which is a result of both the greater separation between the electrode surface and the outer Helmholtz plane and the lower average permittivity of the lipid bilayer compared with interfacial water. The overall shape of the curve is similar to that obtained with other lipids, with a step corresponding to the adsorption/desorption process between around −0.4 and –0.8 V and a change in capacitance at more positive potentials, which suggests a phase transition or the incorporation of electrolyte (which would raise average permittivity). The hysteresis between the negative-going and positive-going sweeps may be a result of slow kinetics of adsorption and desorption, particularly if there are strong intermolecular interactions. The minimum value of specific capacitance in the positive-going sweep is 6.5 μF cm^–2^ and in the negative sweep it is 5.7 μF cm^–2^, at approximately −0.1 V. These values are similar to those measured for DPPC on Au(111) in our earlier study (8 and 5 μF cm^–2^)^56^ and DOPC bilayers on Hg (5 μF cm^–2^).^76^ Using eq 2, it is possible to estimate a coverage of the surface with SM, θ:^77^
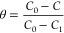
2where *C*_0_ is the capacitance of the clean surface, *C*_1_ is the capacitance of a perfect bilayer, and *C* is the measured capacitance. The specific capacitance of a perfect bilayer may be taken as half that for a lipid monolayer on mercury (the liquid surface of mercury means that defects are not induced by the surface), 0.8 μF cm^–2^.^34^ The value for the bare Au(111)|electrolyte interface at −0.1 V is around 24.8 μF cm^–2^. Using these values for SM gives an estimated coverage of around 80%, which is similar to the 83% obtained for DPPC.^56^ Below, we shall see that this compares well with values obtained from analyzing AFM images of an SM bilayer.

Figure 3b shows chronocoulometry data measured for an SM bilayer. The inset to the figure shows the surface pressure plotted against potential, where surface pressure is given by eq 3.
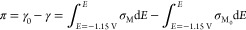
3

A step in the charge density occurs at ca. −0.8 V; the magnitude of the charge density then decreases smoothly until around 0.1 V, where another change in slope occurs. The first step is likely to correspond to the adsorption/desorption of the bilayer and the second change in slope to a phase transition. The overall shape of the charge density–potential plot is slightly different from those of other zwitterionic lipids as the charge density increases at 0.1 V rather than continuing and crossing the curve obtained in the base electrolyte. This behavior is reflected in the plot of surface pressure, which does not pass through a maximum but instead starts to level off at positive potentials. The charge densities for an SM-coated surface are larger than those obtained for DPPC-coated surfaces. One might infer from this observation that the coverage of the surface is lower, but the slope of the charge density–potential plot is very similar, and the coverages estimated from differential capacitance measurements were also close in value. A different orientation of headgroups in the bilayer or interaction of lipids with water may also explain the difference in charge densities. Therefore, AFM was used to determine whether or not coverage of the surface with SM was similar to that previously determined for DPPC.

### 3.3 Atomic Force Microscopy

**Figure 4:**
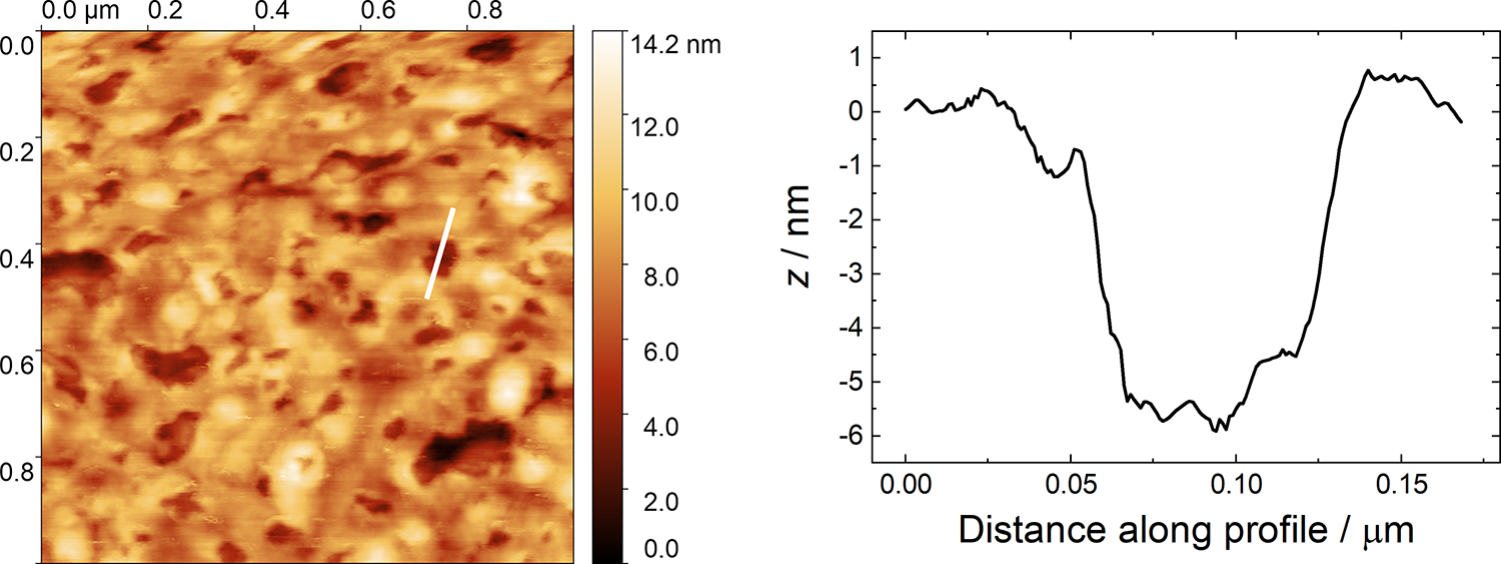
(Left) Representative AFM image of a SM bilayer on a gold-on-glass slide with a depth profile across a defect marked in white; (Right) *z*-axis deflection of the cantilever across the profile marked in the image.

AFM experiments were carried out to determine the coverage of a Au surface with SM as well as the average thickness of the bilayers to facilitate the interpretation of IR spectra (vide infra). A representative image of an SM bilayer on Au is presented in Figure 4, along with a height profile of the marked defect. Further examples of images are available in the Supporting Information (Figure S3). A number of height profiles in images acquired for three samples were measured, and the mean depth was found to be 5.4 nm (with a standard deviation of 0.9 nm). Histograms are provided in the Supporting Information (Figure S4). This thickness is the same as measured for DPPC in a previous study^56^ and is within error of twice the monolayer thickness determined with XRR (5.0 nm). The lower tilt angle of the chains in the monolayer does not have a significant impact on the overall bilayer thickness, as was observed for the overall monolayer thickness from XRR for the two molecules. The images were binarized (as described previously for DPPC bilayers^56^ and DMPC-containing bilayers^78^), and an example of a resulting image is included in the Supporting Information (Figure S5). The average coverage from the binarized images was determined as 85%. This value is in good agreement with the value estimated from capacitance measurements and is similar to that for DPPC bilayers,^56^ which may result from the fact that the substrate–headgroup interactions are the same in each case.

### 3.4 Infrared Measurements

#### 3.4.1 C–H Stretching Modes

**Figure 5:**
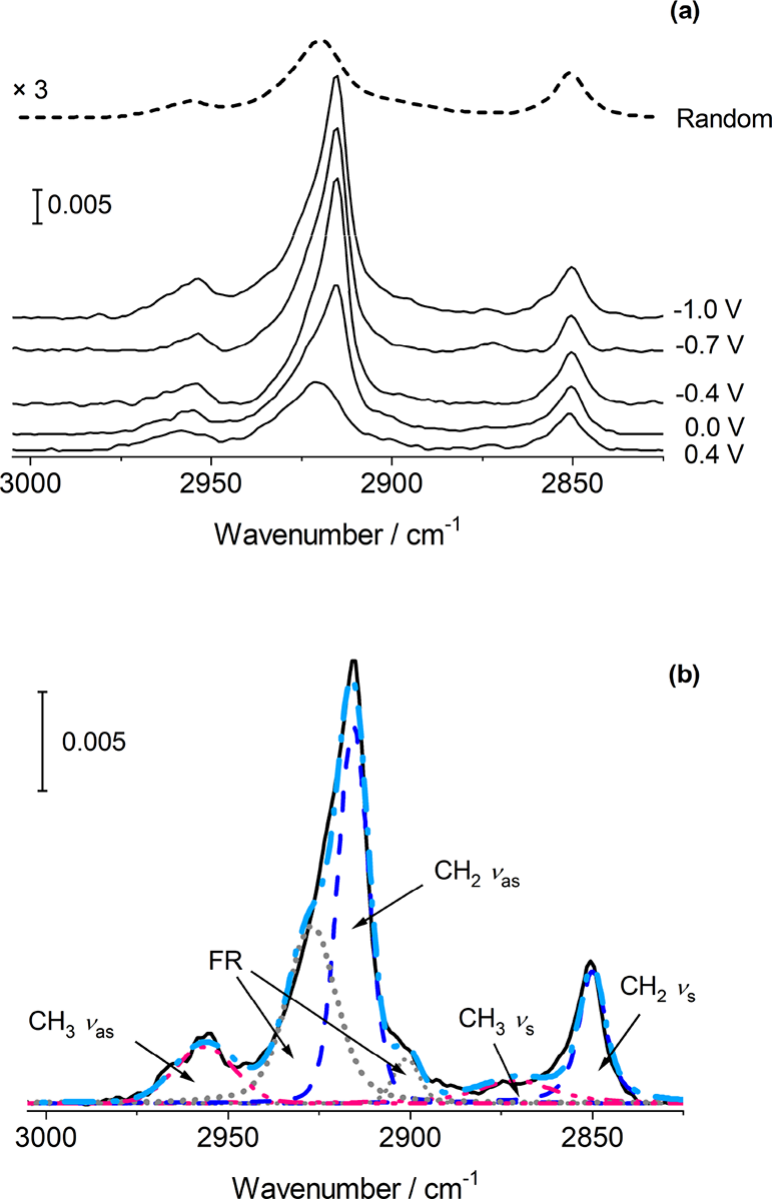
(a) Spectra in the C–H stretching region for a SM bilayer on Au(111) at selected potentials. (b) Example of the deconvolution of a spectrum acquired at 0.0 V.

Spectra of the SM bilayers acquired in the C–H stretching region are presented in Figure 5. As for other lipids, six peaks are found in this region: the methyl symmetric and asymmetric stretching at ∼2870 and 2960 cm^–1^, respectively, the methylene group symmetric and asymmetric stretching at ∼2850 and 2920 cm^–1^, respectively,^27,79−84^ and two Fermi resonances that arise from the combination of the methylene stretching mode with overtones of the methylene bending modes.^81,83,85^ SM has been reported to have bands in similar positions and another at 3007 cm^–1^, corresponding to the olefinic C–H stretching vibration,^84^ but this band was not apparent in our spectra; the intensity was likely too low, perhaps if the transition dipole moment is oriented close to parallel to the surface. The spectra were fitted to these six peaks using a mixed Gaussian–Lorentzian lineshape. The symmetric stretching mode has a roughly constant wavenumber of around 2850–2851 cm^–1^ over the potential range studied and a full width at half-maximum (FWHM) of ∼8 cm^–1^. For the asymmetric stretching vibration, a small decrease in wavenumber from ∼2920 to ∼2917 cm^–1^ was observed over the potential range between 0.45 and 0 V, along with a decrease in the FWHM from around 15 cm^–1^ to around 12 cm^–1^. This potential range corresponds to the range over which the second phase is observed in the electrochemistry measurements. The band center and FWHM are related to the degree of ordering within the chains and their mobility.^79−82^ The wavenumbers of the peaks are consistent with lipids in the gel phase^27,79−82^ and are lower than those reported for DPPC^56^ and DMPC^29^ under similar conditions. The wavenumber is related to the number of *gauche* conformers in the hydrocarbon chains. The values obtained for SM indicate that there are relatively few *gauche* c onformers, with lipid chains predominantly all-*trans*. The FWHM are also low, which indicates low mobility of lipids. Both these observations are consistent with a model of closely packed molecules with strong hydrogen bonding interactions between them.

The spectra in Figure 5 show that the intensities of the two methylene stretching vibrations increase as the potential is made more negative. The intensities of the bands can be used to provide information on the tilt angle of the chains with respect to the surface normal, θ (eq 4).^29,65^
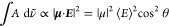
4

The tilt angle, θ, of a transition dipole can be determined by comparing the intensity of the band with that in a theoretical spectrum simulated for a film of the same thickness but with randomly oriented molecules using eq 5.^29,65^
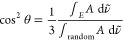
5

The orientation of the chain is related to the orientations of both of the transition dipoles through eq 6:^29,86^
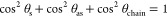
6

**Figure 6:**
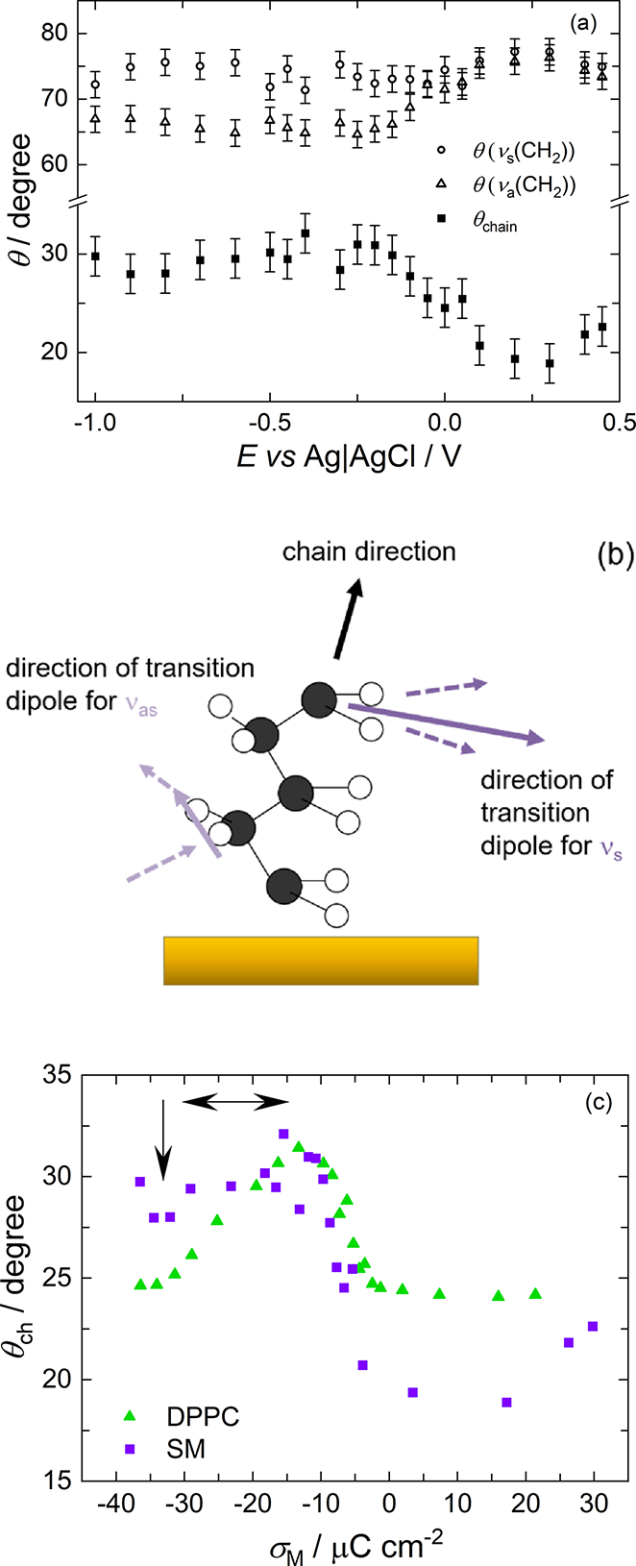
(a) Tilt angles from surface normal of the methylene symmetric (open circles) and asymmetric (open triangles) stretching mode transition dipoles and of the chain (filled squares). (b) Cartoon depicting the directions of the dipoles. (c) Comparison of the chain tilt angles of SM (squares) and DPPC (triangles). Data for DPPC were obtained from ref (56). The double-headed arrow marks the range of the step in charge density (where detachment of the bilayer occurs), and the vertical arrow marks the completion of desorption.

The tilt angles of the transition dipoles and the resulting chain tilt angle are plotted as a function of potential in Figure 6a. Figure 6b shows a cartoon indicating the directions of the transition dipoles with respect to the chain backbone. The chain tilt angle increases as the potential is made more negative, with the change beginning at the point where the slope changes in the charge density–potential plot. At positive potentials, where the surface has low charge density, the tilt angle is around 19°. It rises and goes through a small maximum at around −0.4 V before decreasing to a value of approximately 30° at negative potentials. This decrease takes place over the range where the bilayer is detaching from the surface. Figure 6c compares the chain tilt angles of SM with the previously reported values for DPPC. The SM chains are less tilted at low charge density than for DPPC and are more tilted in the detached bilayer. The tilt angle around the maximum is similar for SM and DPPC, and the tilt angle at the positive potential limit (a slightly positively charged surface) is also similar.

The values of the tilt angle at low charge densities compare very well with those obtained from GIXD data and suggest that the structure of the transferred monolayer is comparable with that on the aqueous subphase. A smaller tilt angle for SM than for DPPC is consistent with the smaller molecular area determined from the isotherm and GIXD measurements and indicates that SM molecules pack more closely than DPPC molecules in the condensed phase. The closer packing is likely to result from intermolecular hydrogen bonding between SM molecules.

#### 3.4.2 Headgroup Vibrational Modes

**Figure 7:**
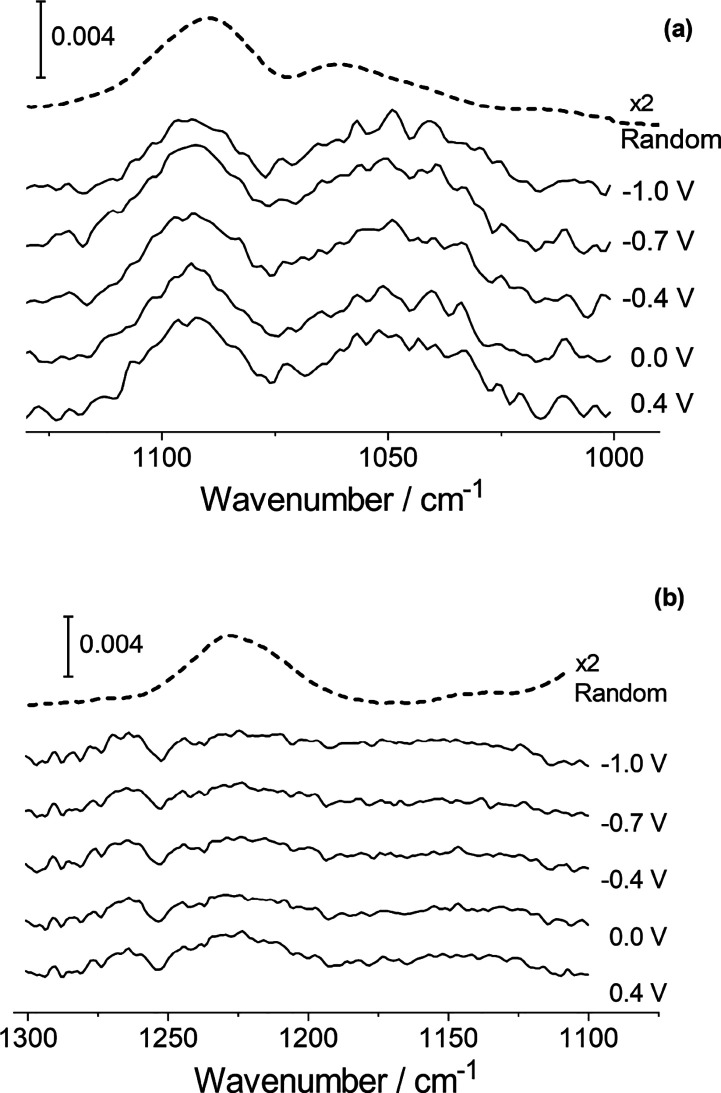
(a) Phosphate symmetric stretching modes at selected potentials. (b) Phosphate asymmetric stretching modes at selected potentials.

Spectra in the phosphate stretching vibration region are presented in Figure 7. Two modes are observed for the unesterified oxygen atoms O–P–O stretching: one at around 1100 cm^–1^ and another at around 1220 cm^–1^. The lower wavenumber mode corresponds to the symmetric O–P–O stretch, and the higher wavenumber mode corresponds to the asymmetric stretch.^27,73,84,87^ The symmetric stretch overlaps with [C]O–P stretching vibrations. For sphingomyelins, these typically appear at ∼1050–1060 cm^–1^.^27,73^Figure 7a shows the spectra in this region. The O–P–O stretching vibration appears at 1094.2 (±0.7) cm^–1^, and another broad band is observed at ∼1050 cm^–1^. This band appears to consist of two components at ∼1040 and 1060 cm^–1^. Splitting of this band has also been reported for egg-SM by Arsov and Quaroni,^27^ who suggested it may indicate intramolecular interactions of the phosphate groups. The wavenumber of the O–P–O symmetric stretching mode is higher than previously reported by de la Arada et al. for egg-SM (1086 cm^–1^)^73^ but, similarly to their observations, it is lower than previously obtained for DPPC under similar conditions.^56^ The wavenumber is typical of relatively dehydrated phospholipid headgroups,^29^ and the spectra bear a strong resemblance to those acquired for DMPS bilayers on Au(111).^53^Figure 7b shows the spectra obtained for the asymmetric O–P–O stretching mode. The wavenumber of the O–P–O asymmetric stretching vibration is 1222.5 (±1.0) cm^–1^, lower than is typical for DPPC but typical for SM.^73^ The low wavenumber suggests that phosphate groups are participating in hydrogen bonding. These spectra also show a mode at ∼1262 cm^–1^, which is probably a methylene wagging mode, as seen with DPPC bilayers,^56^ or possibly an amide III mode (although the latter is normally weak in IR spectra and the band was also visible for DPPC, which contains no amide group). However, as poorly solvated phosphate groups normally absorb around 1240–1255 cm^–1^ and a loss of symmetry in the phosphate group might cause band splitting, assignment of the band at 1262 cm^–1^ to asymmetric phosphate stretching of a population of unsolvated phosphate groups cannot be excluded. Neither the asymmetric nor the symmetric stretching vibrations change significantly with applied potential; the wavenumbers and widths exhibit no trend, and the peak areas decrease slightly, suggesting that the dipole moments become slightly flatter to the surface. The tilt angles of the transition dipoles from the surface normal can be calculated, and eq 6 can be used to calculate the tilt angle of the R–O–P–O–R′ backbone, similarly to the method used for hydrocarbon chains.^29^ Using this method, tilt angles in the range of 23–25° are obtained, smaller than for DPPC^56^ but similar to DMPC.^29^ The tilt angle appears to decrease slightly as the potential is made more negative, but the changes are very small compared with the experimental error. The data overall suggest the phosphate groups’ environment changes little as the molecules reorient, but the wavenumbers of the bands indicate some hydrogen bonding, which could be intramolecular (e.g., with the hydroxyl group) or with neighboring lipid molecules or with water. If interactions between lipid molecules or with water change as the molecules reorient, the effects on the phosphate spectra are similar. Although the change in phosphate group orientation is small, a slight flattening of headgroups is consistent with a decrease in the chain tilt angle. The disparity in the extent of the change in phosphate group orientation and change in chain tilt angle might suggest that the latter is related in part to a headgroup reorientation and in part to a change in conformation where the chains are connected to the headgroup portion of the molecule.

#### 3.4.3 Amide Vibration Modes

**Figure 8:**
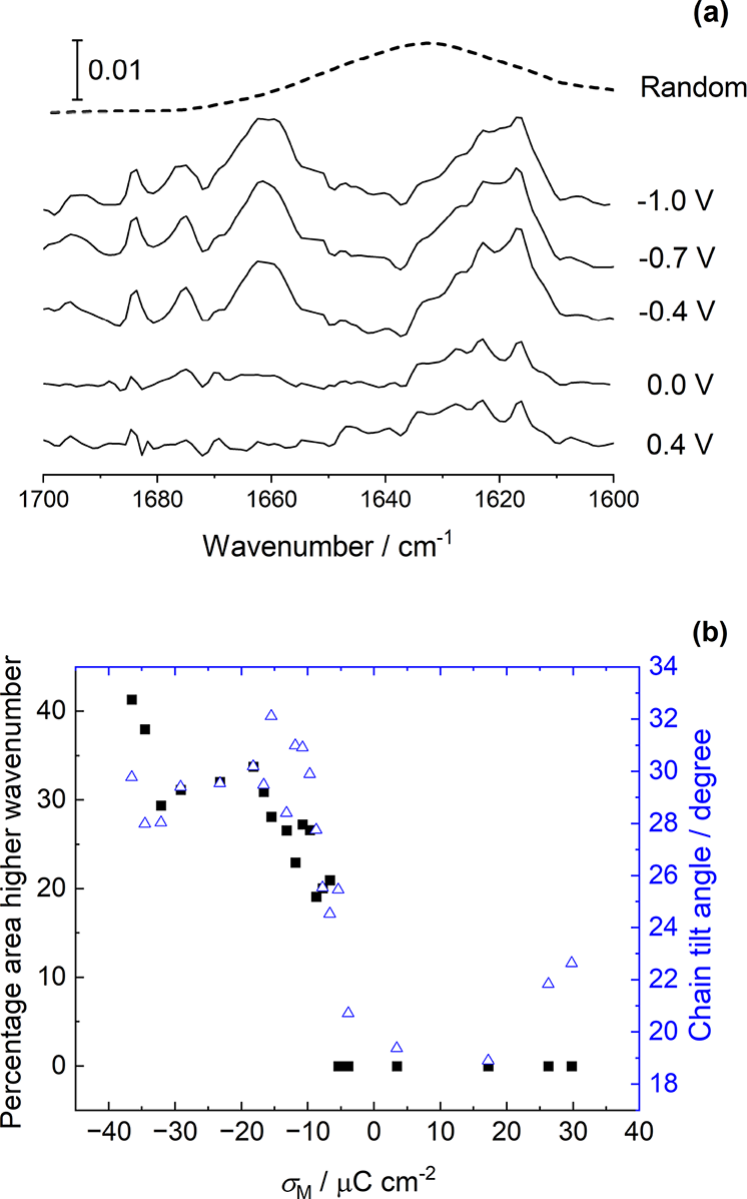
(a) Selected spectra in the amide I region. (b) Proportion of the total area corresponding to the higher wavenumber band (squares) plotted as a function of charge density along with the chain tilt angle (triangles).

Figure 8 presents spectra of the SM bilayer in the amide I region. The main contribution to the amide I absorption is the C=O stretching vibration.^25,26^ The position of the band is sensitive to the hydrogen bonding interactions of the amide group with other molecules.^25−27,73,88,89^ At positive potentials, a broad peak is observed at ∼1630 cm^–1^, which increases in intensity as the potential is made more negative. It appears to be composed of two peaks, the lower wavenumber component dominating at negative potentials. As the intensity of the peak increases, a second peak begins to emerge at a higher wavenumber, ∼1660 cm^–1^. Both peaks correspond to amide I modes, and each corresponds to an amide group in a different environment. A band at ∼1440 cm^–1^ begins to appear at the same potential as the 1660 cm^–1^ band but is weak and overlaps with the envelope of the CH_2_ scissoring mode and headgroup choline modes (Figure S10). The 1440 cm^–1^ band is likely to be the amide II mode, which has contributions from the in-plane N–H/D deformation and associated C–N stretching. This mode is normally observed at around 1550 cm^–1^^25,84^ but is significantly shifted in D_2_O because of the higher reduced mass. The spectra in this region were noisy and difficult to fit, so they were not analyzed further.

Spectral features associated with the amide I mode in sphingolipids usually comprise more than one band. The higher wavenumber component of the amide I mode has previously been assigned to a desolvated amide group, and the lower wavenumber component to a more solvated amide group^88^ or, more generally, to those SM molecules weakly hydrogen bonding (or not hydrogen bonding) and those whose carbonyl groups are involved in hydrogen bonding.^27^ Studies of IR spectra through the chain-melting phase transition of chicken egg-SM (predominantly SM with palmitoyl chains) show a gradual increase in wavenumber with a jump to alower wavenumber at the phase transition temperature, which was attributed to a change in solvation and/or a conformational change as the SM changes from the gel phase to the liquid crystalline phase.^89^ Raman spectra show a strong band at 1689 cm^–1^ assigned to the C=C stretch (which is weaker in IR spectroscopy and is not observed in our spectra) and a band at 1643 cm^–1^, which has been assigned to the amide I mode.^25,26^ A detailed study of molecular dynamics and simulation of the IR and Raman spectra of the amide portions of SM molecules has shed further light on the effects of different hydrogen bonding interactions on the composition (and, thus, shape) of this band.^25,26^ The band was shown to be a composite of bands with different wavenumbers, each corresponding to SM molecules involved in different hydrogen bonding interactions, some as monomers and some as dimers or small groups. These results showed that the lower wavenumber components (∼1643 cm^–1^) were related to carbonyl groups where the oxygen atom accepted two hydrogen bonds, while higher wavenumbers corresponded to carbonyl groups accepting one hydrogen bond (or still higher, to those accepting no hydrogen bonds). Further studies showed that the calculated position of the amide band shifted to lower wavenumber as the number of interacting SM molecules in a ″cluster″ was increased and explored the dependence of the experimentally observed 1643 cm^–1^ band on hydration and on mixing with other components. These results demonstrated that the presence of the band could be used as a marker for clusters of SM molecules.^26^ The band increased in intensity on drying a SM sample and disappeared on rehydration or on mixing SM with cholesterol. Further, when di-oleoyl phosphatidylcholine (DOPC) was added to SM samples, the 1643 cm^–1^ band was present, while when DPPC was added to SM, it was absent. As SM is known to be miscible with DPPC but not with DOPC, the presence of the band was taken as a marker for clusters of SM in the DOPC/SM sample.^26^

Taking into account the ∼10 cm^–1^ shift in wavenumber from N–H/D exchange in D_2_O,^25^ our band at 1630 cm^–1^ may be assigned to C=O stretching in clusters of SM interacting via hydrogen bonds, and our band at 1660 cm^–1^ may be assigned to C=O stretching in SM molecules where the carbonyl group accepts one hydrogen bond (from water or perhaps another amide group). Both bands increase in intensity at negative potentials because the C=O transition dipole moment is perpendicular to the hydrocarbon chains, which are more tilted at negative potentials. The intensity cannot be used to calculate a tilt angle because of the changes in hydrogen bonding, but the proportion of the contribution of the lower wavenumber component to the total amide absorption is clearly seen to decrease at negative potentials, as plotted in Figure 8b. These results could be interpreted as a loss of water from the bilayer, as previously observed for DPPC at the same charge density, reducing the population of SM molecules accepting two hydrogen bonds. They can also be interpreted as a disruption of a hydrogen bonding network among SM molecules, with some clusters of SM molecules breaking up to leave monomers or dimers each interacting with one water molecule. A small change in band center (or increase in proportion of the lower wavenumber component of the band) may indicate a change in conformation of the carbonyl groups of some SM molecules, potentially SM molecules ″freed″ from the network and returning to a closer to planar conformation, but the change is small. The data suggest that on increasing the strength of the applied field, the chains reorient, SM–SM interactions are disrupted, and water leaves the bilayer. The concomitant reorientation of the chains and water egress as the negative charge is increased are similar to the behavior of glycerophospholipids. However, the ability of SM to form multiple direct intermolecular hydrogen bonds means that it initially forms relatively disordered but closely packed networks of molecules, which are then disrupted by the electric field. Whereas the glycerophosphocholine bilayer structure is driven by the chain–chain interactions, the sphingolipid bilayer structure is dominated by the hydrogen bonds between molecules, but the stronger interactions between the molecules do not prevent the disruption of the bilayer or membrane permeabilization by an electric field. Instead, the arrangement of the hydrocarbon chains is changed by the perturbation and, unlike DPPC, the chains cannot return to a similar arrangement on desorption.

## 4 Conclusions

The effect of an applied electric field on the intermolecular interactions between sphingomyelin molecules in a supported bilayer has been studied with a combination of electrochemical methods and in situ PM-IRRAS. AFM was used to determine the coverage of SM on the substrate and matched well the value estimated from differential capacitance measurements. The coverage of SM was very similar to that previously reported for DPPC, a close glycerophospholipid analogue, and the thickness of SM bilayers was also found to be similar to that of DPPC bilayers. GIXD data for monolayers on water show that the average area occupied per SM molecule is smaller than for DPPC because the hydrocarbon chain tilt angle is smaller. The chain tilt angle obtained from GIXD is close to that calculated from the PM-IRRA spectra of the supported bilayers; the relatively low intensity of the reflections indicates a smaller degree of long-range order compared with DPPC and suggests smaller groups of SM molecules. The electrochemical response of SM differs from that of DPPC; instead of a maximum in the surface pressure around the potential of zero charge, the bilayer appears to undergo a phase transition at more positive potentials. PM-IRRAS data showed that this second transition is related to a small increase in hydrocarbon chain tilt angle: unlike the glycerophospholipids so far reported, SM chain tilt angles increase either side of the potential of zero charge. As the surface charge density becomes more negative, SM chains tilt farther from the surface normal, as has been observed for DPPC and DMPC, but remain tilted at the most negative charge densities, as has been observed previously for the anionic lipid DMPS.^53^ This behavior is correlated with a change in the amide vibrational modes, which show a change in solvent content of the bilayers as the potential is made more negative and water interacts with the field. The spectra in the amide region indicate that SM forms small clusters within the bilayer and that the increasing field and solvent content disrupt this hydrogen bonding network, with the SM–SM hydrogen bonds replaced with SM–water hydrogen bonds. SM and DPPC are very similar in structure, with similar chain lengths and the same headgroup; they differ only in the linkage of their chains to the headgroup, yet that small structural change has profound effects on how the molecules interact and respond to external stimuli. The results from this study shed light on how sphingomyelin and other related sphingolipids behave in fields comparable with those found in natural cell membranes and how this behavior may differ from their glycerophospholipid analogues. This has particular importance both for the structural roles of sphingolipids in rafts in maintaining the local structure and tension around *trans*-membrane proteins and for their multiple roles in cell membrane processes, such as endocytosis and cell signaling.

## Notes

All data supporting this publication are openly available from the UBIRA eData repository at https://doi.org/10.25500/edata.bham.00000863.

## Supporting Information Available

Surface pressure-area isotherm, X-ray reflectivity data and analysis, additional AFM images and analysis, and additional IR spectra and analysis (PDF)
