# Supplementary material for: Influence of the Lipid Backbone on Electrochemical Phase Behavior
Source: Langmuir. 2022 Nov 10;38(46):14290–301. doi: 10.1021/acs.langmuir.2c02370 (PMC9686133; doi:10.1021/acs.langmuir.2c02370)
Supplement: Supplementary file 1 — la2c02370_si_001.pdf [file la2c02370_si_001.pdf]

Influence of the Lipid Backbone on Electrochemical Phase Behavior

Electronic Supporting Information

Philip N. Jemmett,[1] David C. Milan,[2] Richard J. Nichols,[2] Thomas Howitt,[1] Alexandra L. Martin,[1] Thomas Arnold,[3,4,5,6] Jonathan L. Rawle,[3] Christopher L. Nicklin,[3] Timothy R. Dafforn,[7] Liam R. Cox[1] and Sarah L. Horswell[1,*]

[1]School of Chemistry, University of Birmingham, Edgbaston, Birmingham, B15 2TT, UK

[2]Department of Chemistry, University of Liverpool, Crown Street, Liverpool, L69 7ZD, UK

[3]Diamond Light Source, Harwell Science and Innovation Campus, Chilton, Didcot, Oxfordshire, OX11 0DE, UK

[4]European Spallation Source ERIC PO Box 176, SE-221 00 Lund, Sweden

[5]ISIS Pulsed Neutron and Muon Source, Science and Technology Facilities Council, Rutherford Appleton Laboratory, Harwell, Oxfordshire, OX11 0QX, UK

[6]Department of Chemistry, University of Bath, Claverton Down, Bath, BA2 7AY, UK

[7]School of Biosciences, University of Birmingham, Edgbaston, Birmingham, B15 2TT, UK

*Corresponding author: s.l.horswell@bham.ac.uk

1. Langmuir isotherm data

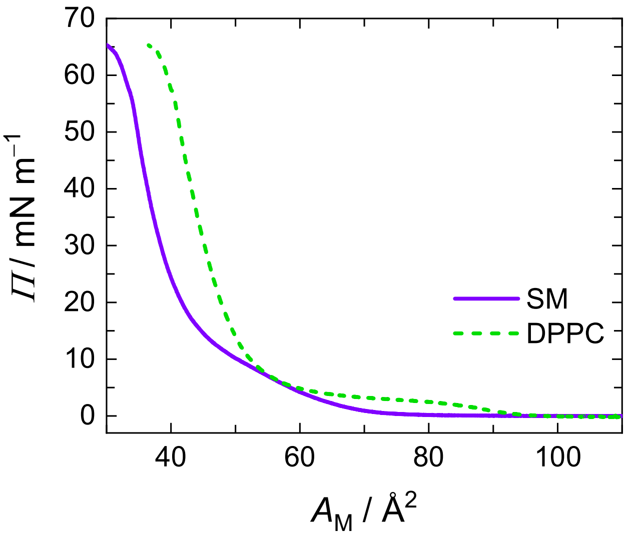

Figure S1. Comparison of isotherms of SM (solid line) and DPPC (dashed line) at 18 °C. Barrier speed 25 cm^2 min^{-1} (maximum trough area 600 cm^2). Data for DPPC taken from ref. S1.

2. X-ray Reflectivity

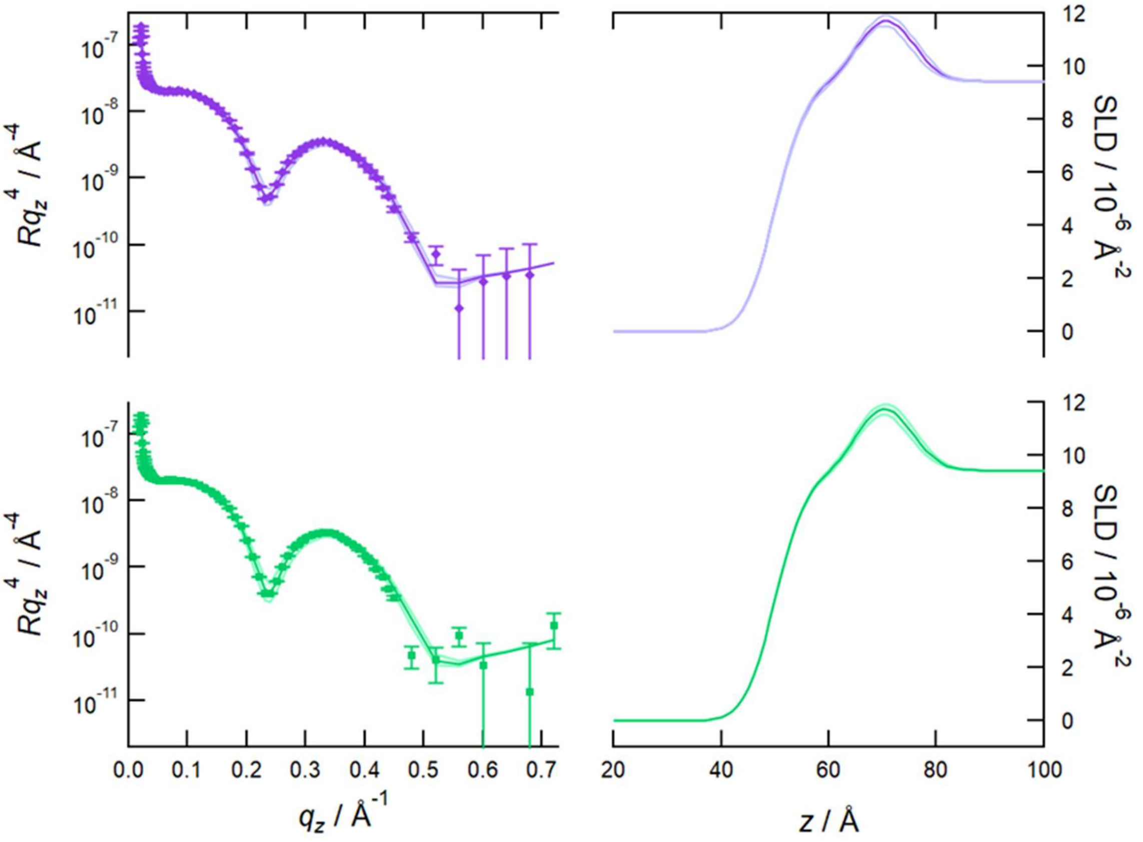

Figure S2. Left: Comparison of X-ray Reflectivity data for SM (top, purple) and DPPC (bottom, green) at 40 mN m^{-1}. Right: Corresponding scattering length density profiles. Shaded regions represent the 95% confidence range.

The data were fitted to a two-slab model, one comprising the tailgroups and the other the headgroups, using RasCAL 2019.[S2] The roughness at each interface was kept the same and at fixed values. A series of fits was carried out for different values of roughness between 3 Å and 5 Å and the tailgroup SLD and thickness were used to calculate the area per molecule (A_M). The closeness of the area per molecule to that determined from diffraction measurements was used along with the quality of the fit itself to select the best fit to the data.

The area per molecule was kept the same for the headgroup slab and was used with the thickness to calculate the solvated headgroup volume. This volume and the SLD of the headgroup slab were used to derive unsolvated headgroup volume and water content. In this way, assumptions about the unsolvated headgroup volume were avoided but the requirement for the Gibbs excess to be the same in each slab was maintained. Table S1 gives the parameters obtained from the fit.

Table S1. Parameters determined from the fits to the data in Figure S2. The numbers in brackets in the second to fourth columns refer to the 95% confidence range from the fit. Those in the last four columns are estimated resulting errors. (σ represents roughness and was fixed. V refers to molecular volume of the slab indicated by the subscript. d refers to slab or monolayer thickness, as indicated.)

	σ / Å (fixed)	V_{tail} / Å^3	d_{tail} / Å	d_{hg} / Å	water	V_{hg} / Å^3	Total d / Å	A_M / Å^2
SM (1)	4.5	733 (729–737)	16.2 (16.0–16.5)	8.5 (8.0–8.9)	2.7 (1.6–3.6)	304 (276–326)	24.7 (24.4–24.9)	45.2 (44.5–45.9)
SM (2)	4.45	739 (735–743)	16.3 (16.1–16.6)	8.6 (8.1–9.1)	2.5 (1.4–3.6)	312 (282–383)	24.9 (24.6–25.1)	45.2 (44.5–45.9)
**Ave. SM**	**4.475**	**736**	**16.3**	**8.4**	**2.6**	**308**	**24.8**	**45.2**
DPPC (1)	4.5	746 (742–750)	15.9 (15.6–16.1)	8.6 (8.1–9.1)	2.3 (1.2–3.5)	337 (308–366)	24.5 (24.2–24.7)	47.1 (46.3–47.9)
DPPC (2)	4.45	757 (755–760)	16.3 (16.0–16.7)	8.6 (8.0–9.0)	1.4 (0–2.3)	359 (327–382)	24.8 (24.6–25.2)	46.6 (45.4–47.3)
**Ave. DPPC**	**4.475**	**752**	**16.1**	**8.6**	**1.8**	**348**	**24.7**	**46.9**

3. AFM images and analysis

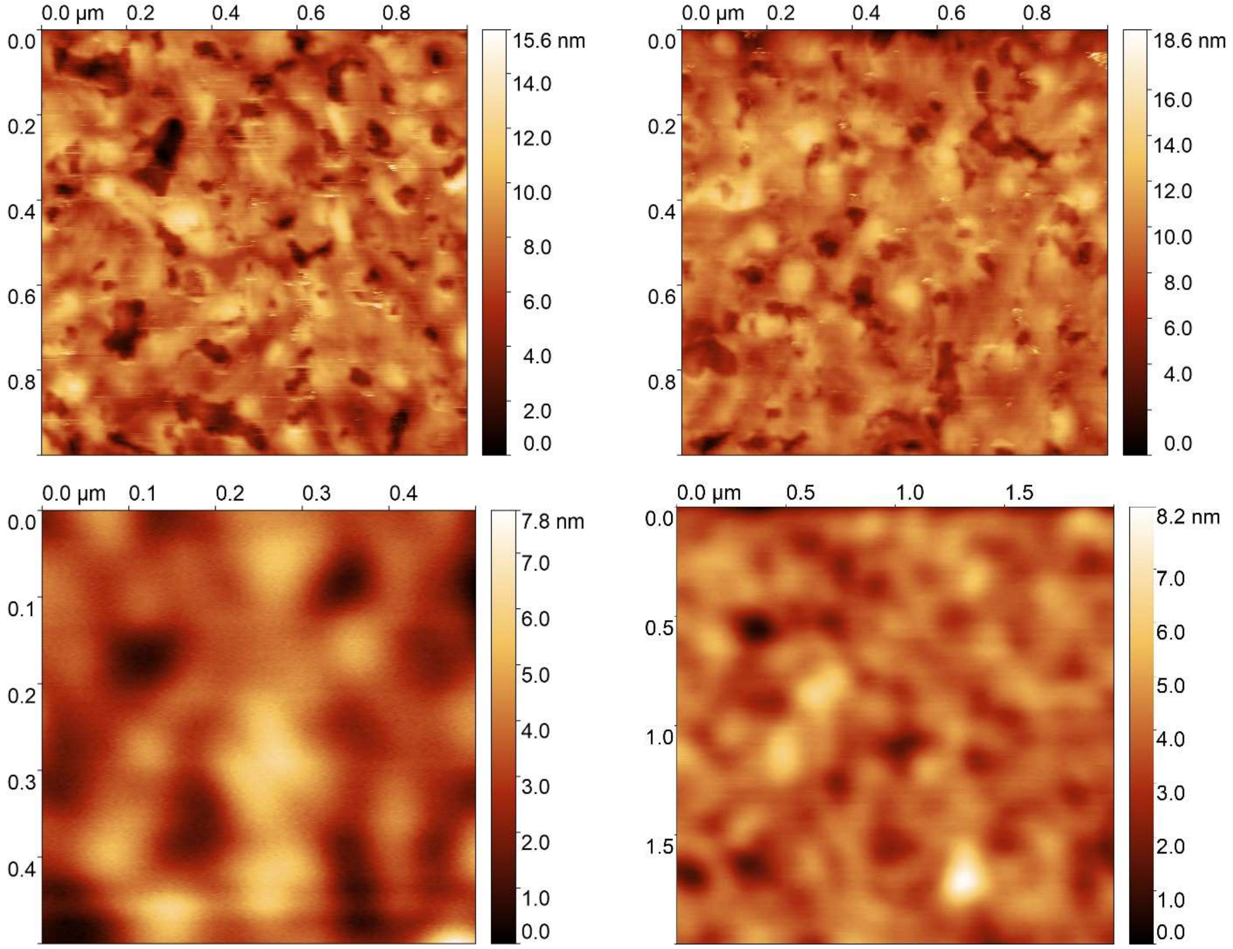

Figure S3. Additional example images of AFM images of SM bilayers on gold-on-glass slides. Images were recorded for different areas of three different samples and height profiles measured across defects. Histograms of the defect depths are presented in Figure S4.

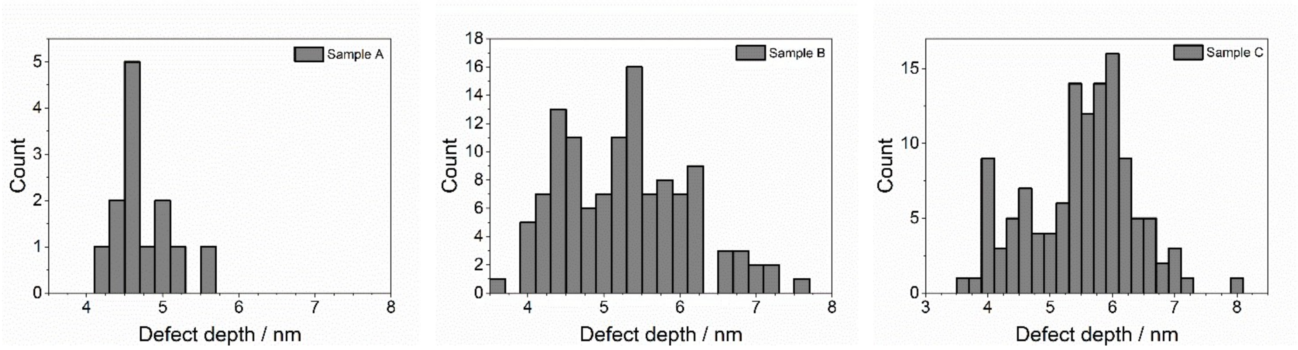

Figure S4. Histograms of the height distributions measured from height profiles of AFM images of SM bilayers on gold-on-glass slides (three different samples). Note that although there is some uncertainty in the height (resulting from differences in elasticity between lipid and substrate), the mean heights are within error of twice the monolayer thickness and the chain tilt angles calculated from IR data and the mean height determined here are in very good agreement with values obtained from GIXD images.

Table S2. Average (mean) bilayer heights determined from the histograms in Figure S4.

Sample	Number of profiles	Average depth / nm	Standard deviation / nm
A	13	4.7	0.4
B	120	5.3	0.9
C	123	5.5	0.9
**Overall**	**256**	**5.4**	**0.9**

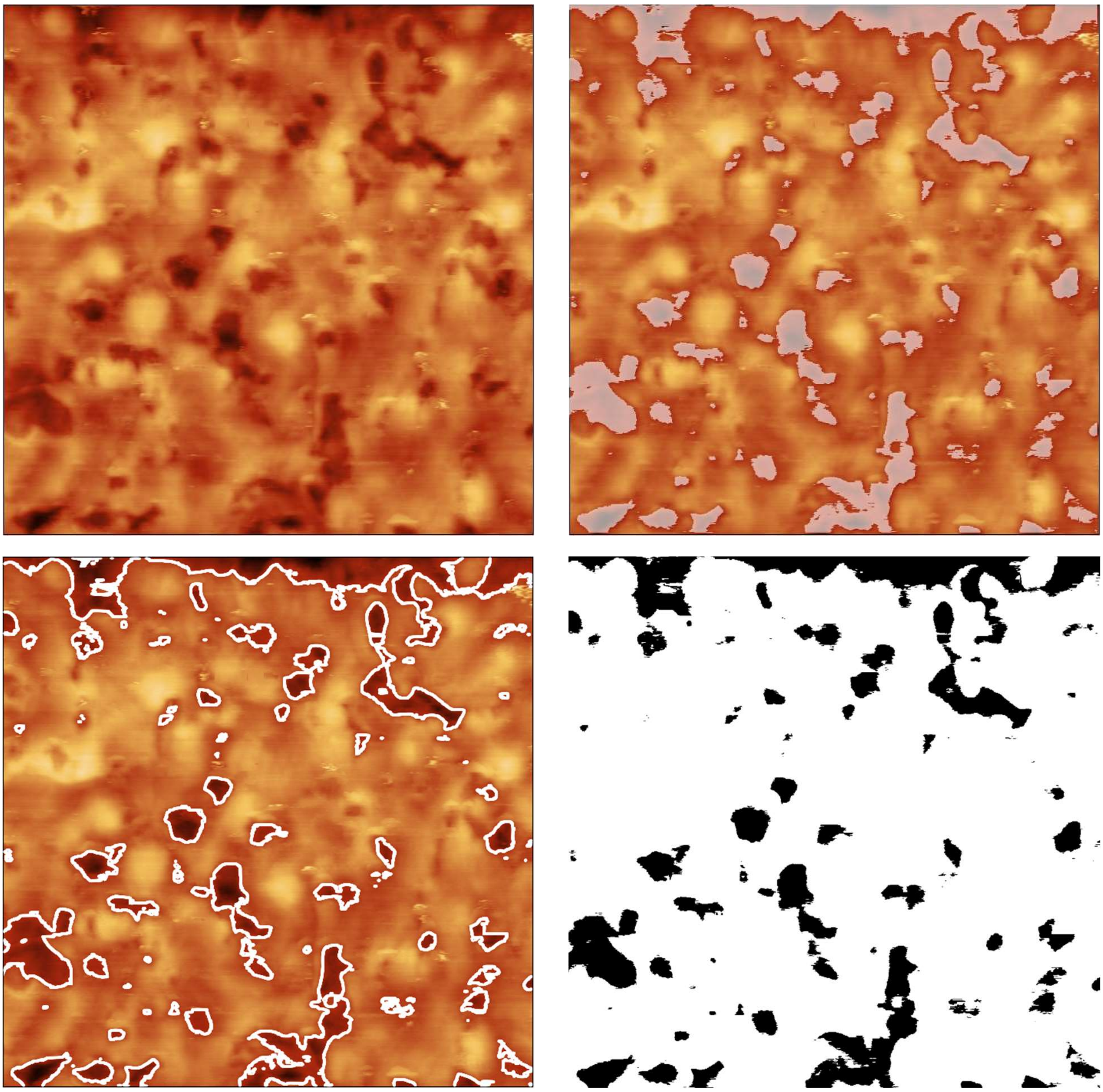

Figure S5. Binarization of AFM images. Top left: original image. Top right: the same image with a false color overlay representing the areas of the slide that are not covered with a SM bilayer. Bottom left: the same image with the edges of the defects highlighted in white. Bottom right: binarized version of the same image with black pixels for areas not covered with lipid and white pixels for areas covered with lipid. The image dimensions are 1 μm × 1 μm.

To generate the binarized form of the image, pixels representing a height of one monolayer or above were assigned values of 1 and all others were assigned values of 0, using a custom-written MATLAB script. The image was then plotted as a binary image and the coverage calculated as a percentage of pixels with value 1.

4. Additional IR Spectra

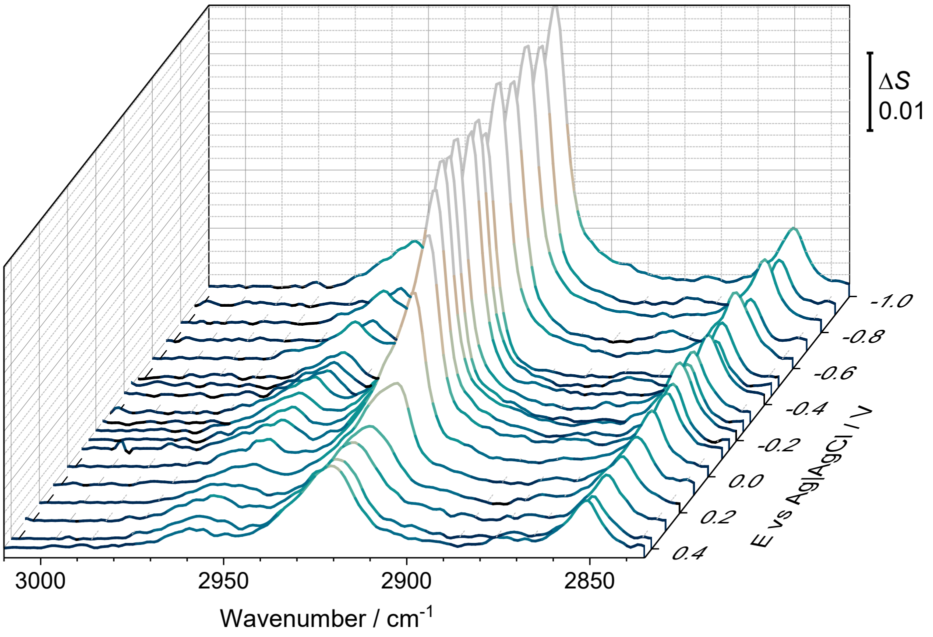

Figure S6. IR spectra in the C–H stretching region, acquired at the indicated applied potentials.

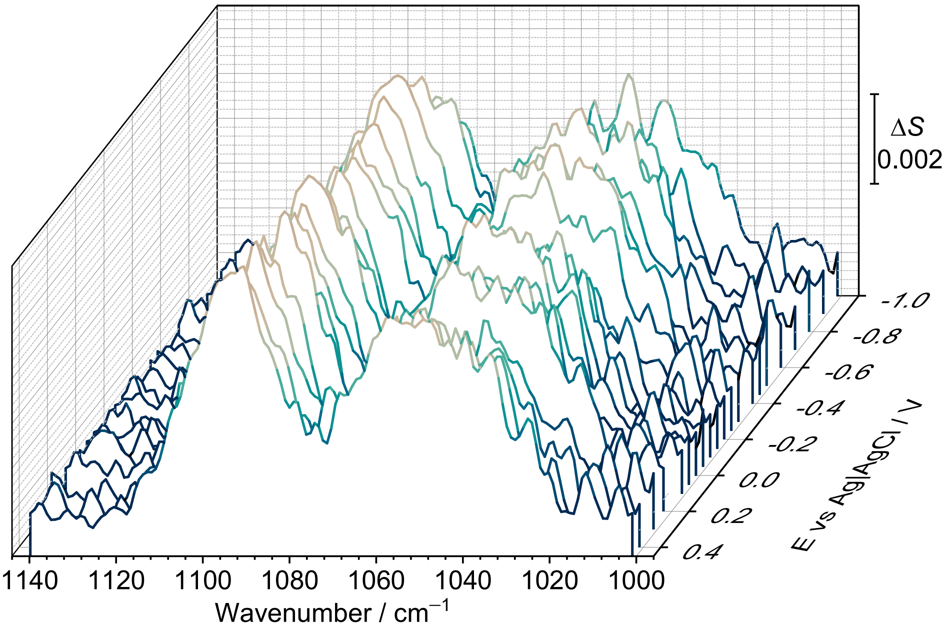

Figure S7. IR spectra in the phosphate (O–P–O symmetric) stretching region, acquired at the indicated applied potentials.

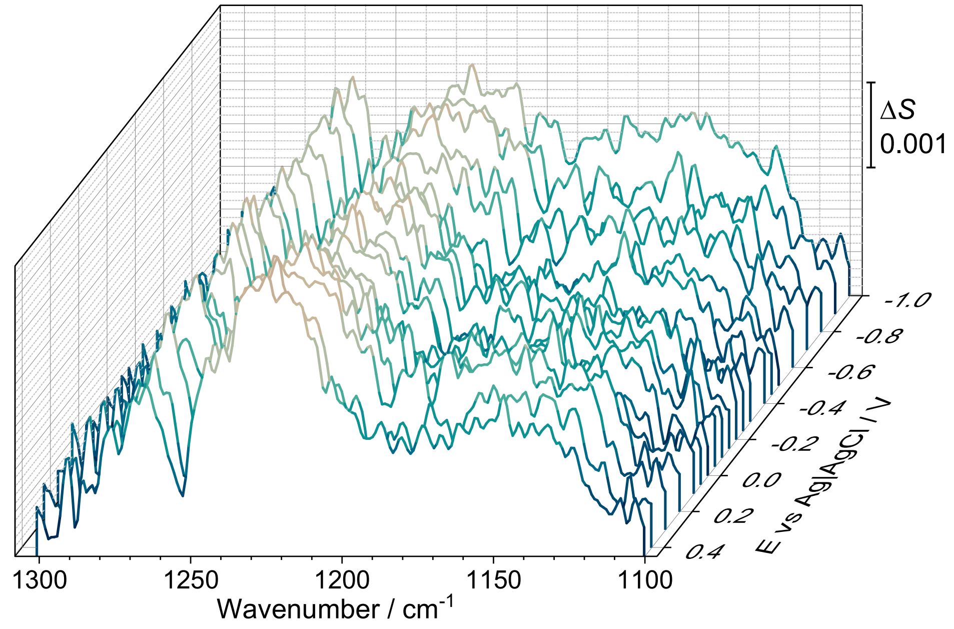

Figure S8. IR spectra in the phosphate (O–P–O asymmetric) stretching region, acquired at the indicated applied potentials.

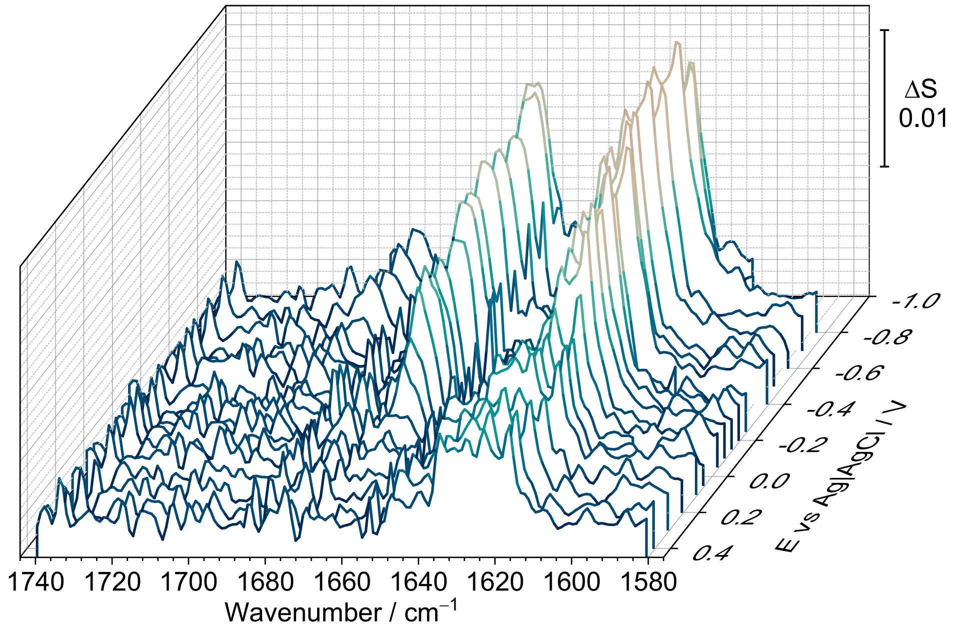

Figure S9. IR spectra in the amide I region, acquired at the indicated applied potentials.

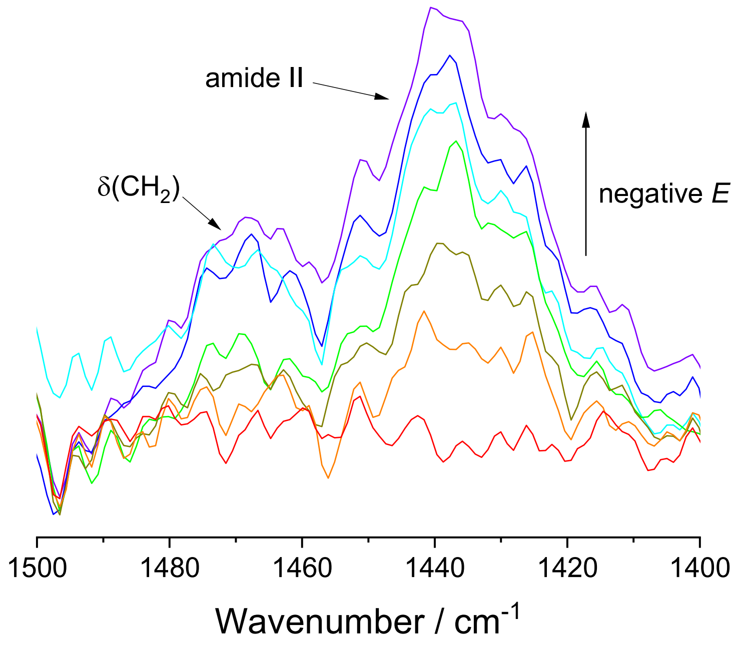

Figure S10. Difference spectra from the amide II region.

$S_{pot} - S_{0.45}$, with S_{pot} at E = 0.4 V, 0 V, –0.1 V, –0.25 V, –0.4 V, –0.7 V, –1.0 V.

The CH_2 scissoring mode increases in intensity with more negative potential, mirroring the increase in intensity of the CH_2 symmetric stretching mode (the change in dipole moment has the same direction). The larger peak is assigned to amide II for a deuterium-exchanged amide group. (The main contributions to this mode are N–D bending and associated C–N stretching.) The increase in intensity of this peak mirrors the increase in intensity of the amide I mode, which suggests the plane containing the amide group changes its tilt angle.

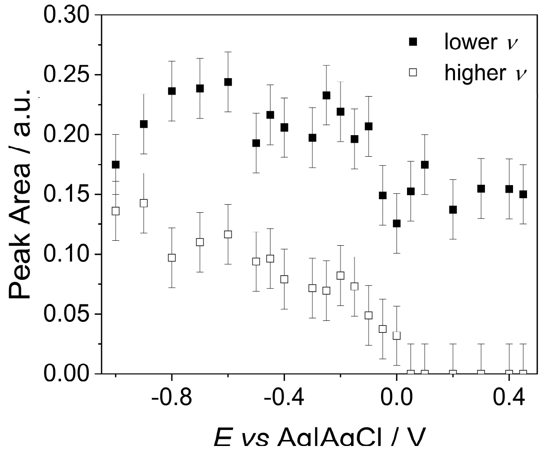

Figure S11. Comparison of the integrated intensities of the two amide I modes as a function of potential.

References

S1. Jemmett, P. N.; Milan, D. C.; Nichols, R. J.; Cox, L. R.; Horswell, S. L. Effect of Molecular Structure on the Electrochemical Phase Behavior of Phospholipid Bilayers on Au(111). *Langmuir* **2021**, *37*, 11887−11899.

S2. Hughes, A. V. RasCAL_2019. https://github.com/arwelHughes/RasCAL_2019
